# Structure, kinetics, and mechanism of *Pseudomonas putida* sulfoquinovose dehydrogenase, the first enzyme in the sulfoglycolytic Entner-Doudoroff pathway

**DOI:** 10.1042/BCJ20240605

**Published:** 2025-01-22

**Authors:** Laura Burchill, Mahima Sharma, Niccolay Madiedo Soler, Ethan D. Goddard-Borger, Gideon J. Davies, Spencer J. Williams

**Affiliations:** 1 School of Chemistry and Bio21 Molecular Science and Biotechnology Institute, University of Melbourne, Parkville, Victoria 3010, Australia; 2 Department of Chemistry, York Structural Biology Laboratory, University of York, York YO10 5DD, U.K.; 3 The Walter and Eliza Hall Institute of Medical Research, Parkville, Victoria, 3052, Australia; 4 Department of Medical Biology, University of Melbourne, Parkville, Victoria, 3010, Australia

**Keywords:** enzyme mechanism, organosulfur, short-chain dehydrogenase/reductase, sulfoglycolysis, sulfur cycle

## Abstract

The sulfosugar sulfoquinovose (SQ) is catabolized through the sulfoglycolytic Entner-Doudoroff pathway, beginning with the oxidation of SQ to sulfogluconolactone by SQ dehydrogenase. We present a comprehensive structural and kinetic characterization of *Pseudomonas putida* SQ dehydrogenase (*Pp*SQDH). *Pp*SQDH is a tetrameric enzyme belonging to the short-chain dehydrogenase/reductase (SDR) superfamily with a strong preference for NAD^+^ over NADP^+^. Kinetic analysis revealed a rapid equilibrium ordered mechanism in which the NAD^+^ cofactor is the first substrate to bind, and NADH is the last product to dissociate. Structural studies revealed a homotetrameric structure in solution and crystals, involving cross-subunit interactions in which the C-terminus residue (Gln260) inserts into the diagonally opposite subunit to form part of the second shell of residues lining the active site. Complexes of *Pp*SQDH with SQ or NAD^+^ provide insight into the recognition of SQ and together with the kinetic analysis allow the proposal of a catalytic reaction mechanism. Our findings illuminate the mechanism of SQ degradation and the evolution of the SDR superfamily for organosulfonate catabolism.

## Introduction

Sulfoquinovose (SQ, 6-deoxy-6-sulfoglucose) is a sulfosugar with a structure analogous to D-glucose but containing a sulfonate at carbon-6 *in lieu* of a hydroxyl group. SQ is a component of sulfoquinovosyl diacylglycerol (SQDG), a sulfolipid found in plants, algae, and cyanobacteria [1]. SQDG is also produced by photosynthetic flagellates and some non-photosynthetic bacteria [1]. SQ has an estimated production on the scale of 10 billion tonnes per annum, and thus, its biosynthesis and catabolism are important to the biogeochemical sulfur and carbon cycles [1,2]. Sulfoquinovosidases liberate SQ from SQ glycosides including SQDG and the delipidated species sulfoquinovosyl glycerol (SQGro) [3,4]. SQ is catabolized through bacterial pathways of sulfoglycolysis and SQ sulfolysis [5]. Sulfoglycolytic pathways involve the cleavage of carbon-carbon bonds in the carbon chain of SQ, while sulfolysis pathways involve the cleavage of the sulfur-carbon bond [6,8]. Sulfoglycolysis pathways are dedicated pathways for SQ degradation that mimic glycolysis pathways including the sulfoglycolytic Embden-Meyerhof-Parnas (sulfo-EMP) [9], sulfoglycolytic Entner-Doudoroff (sulfo-ED) [10,11], sulfoglycolytic sulfofructose transaldolase (sulfo-SFT) [12,13] and sulfoglycolytic sulfofructose transketolase (sulfo-TK) [6] pathways.

**Figure 1:**
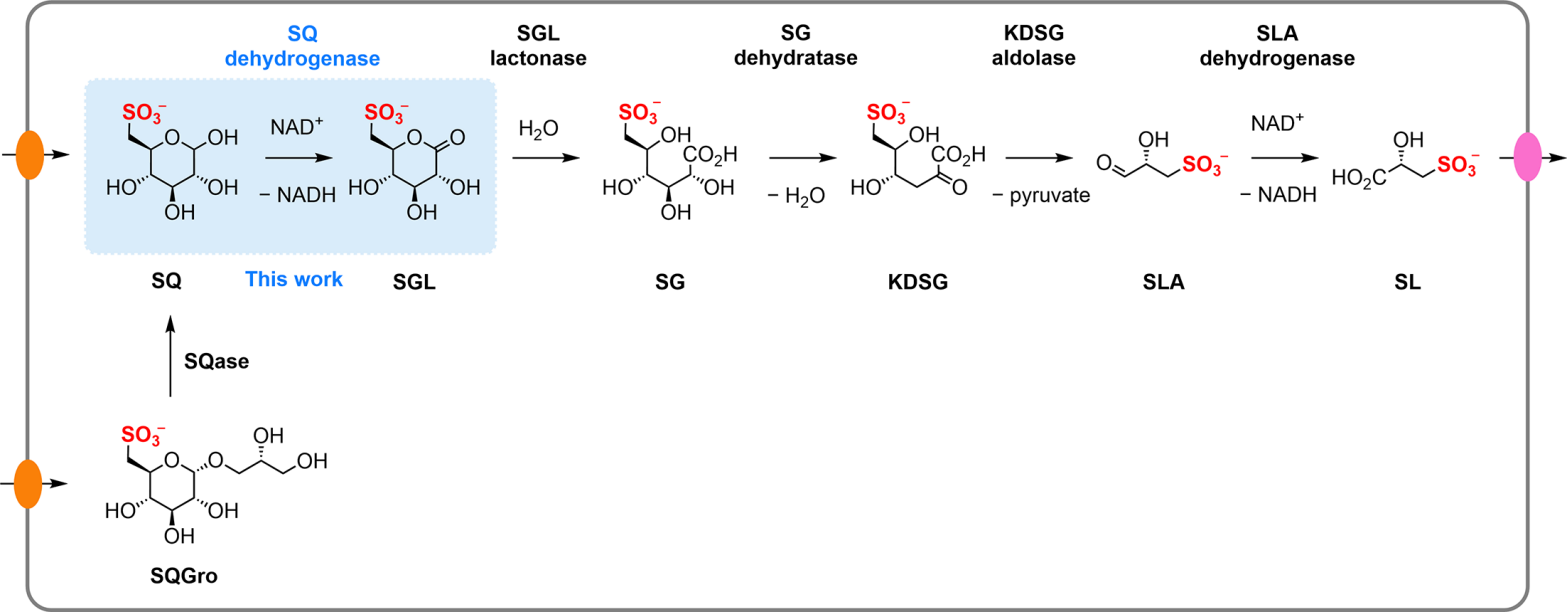
The sulfoglycolytic Entner-Doudoroff pathway in *Pseudomonas putida*, indicating the role of SQ dehydrogenase (*Pp*SQDH).

The first sulfo-ED pathway was described in *Pseudomonas putida* SQ1 [10]. *P. putida* SQ1 was isolated from Lake Konstanz, Germany, through enrichment culture in minimal media using SQ as the sole carbon source [14]. In this pathway, the first step involves the oxidation of SQ to sulfogluconolactone (SGL) through the action of SQ dehydrogenase, a nucleotide cofactor-dependent enzyme. Then, SGL is hydrolyzed by SGL lactonase to give sulfogluconate (SG), which is subsequently converted to 2-keto-3-deoxysulfogluconate (KDSG) by SG dehydratase (Figure 1). The namesake sulfoglycolysis step is catalyzed by KDSG aldolase, which cleaves KDSG into pyruvate and sulfolactaldehyde (SLA). Finally, SLA is oxidized by SLA dehydrogenase [15] to give sulfolactate (SL), which is excreted by the bacterium. Genetic evidence indicates that some bacteria with a sulfo-ED pathway (e.g. *Aeromonas cavernicola*) encode a homolog of SLA reductase instead of SLA dehydrogenase, and thus may produce 2,3-dihydroxypropanesulfonate (DHPS) rather than SL [6]. The resulting short-chain organosulfonates SL and DHPS are substrates for other bacteria that contain degradative pathways allowing complete utilization of their sulfur and carbon content [16,18].

Our expanding knowledge of the pathways for the breakdown of SQ has identified new enzymes, binding proteins, and SQ-degrading bacteria. This has also inspired novel chemical biology tools. In the human gut, sulfoglycolysis is employed by gut bacteria such as *Eubacterium rectale* [12], a *Firmicute* known for producing health-promoting short-chain fatty acids [19]. Sulfoglycolysis of SQ by *E. rectale* generates DHPS, which is metabolized by *Bilophila wadsworthia*, a sulfite-reducing bacterium, to produce H_2_S, a molecule with complex effects on human gut health [19]. SQ import is often mediated by ATP-binding cassette (ABC) transporters and SQ-binding proteins [7], and these have been applied for affinity purification using an SQ-modified agarose resin [20]. Recent interest in sulfoglycolysis has spurred the development of activity-based protein profiling reagents targeting sulfoquinovosidases [3,21]. These findings advance our understanding of sulfoglycolytic pathways and hold promise for applications in human health and biotechnology.

While there is extensive and growing knowledge of the structure and mechanisms of many of the enzymes of other sulfoglycolytic and SQ sulfolytic pathways, comparatively, relatively little is known of the enzymes of the sulfo-ED pathway. Here, we describe a structural and kinetic study of the first pathway-specific enzyme of the sulfo-ED pathway, SQ dehydrogenase, a member of the short-chain dehydrogenase/reductase (SDR) superfamily. We show that this enzyme is highly specific for nicotinamide adenine dinucleotide (NAD^+^) and operates through an ordered kinetic mechanism in which NAD^+^ binds to the enzyme first. Structures of the enzyme in complex with SQ or NAD^+^, determined using X-ray crystallography, allow the proposal of a catalytic reaction mechanism.

## Results

### Kinetic analysis of SQ dehydrogenase

**Figure 2:**
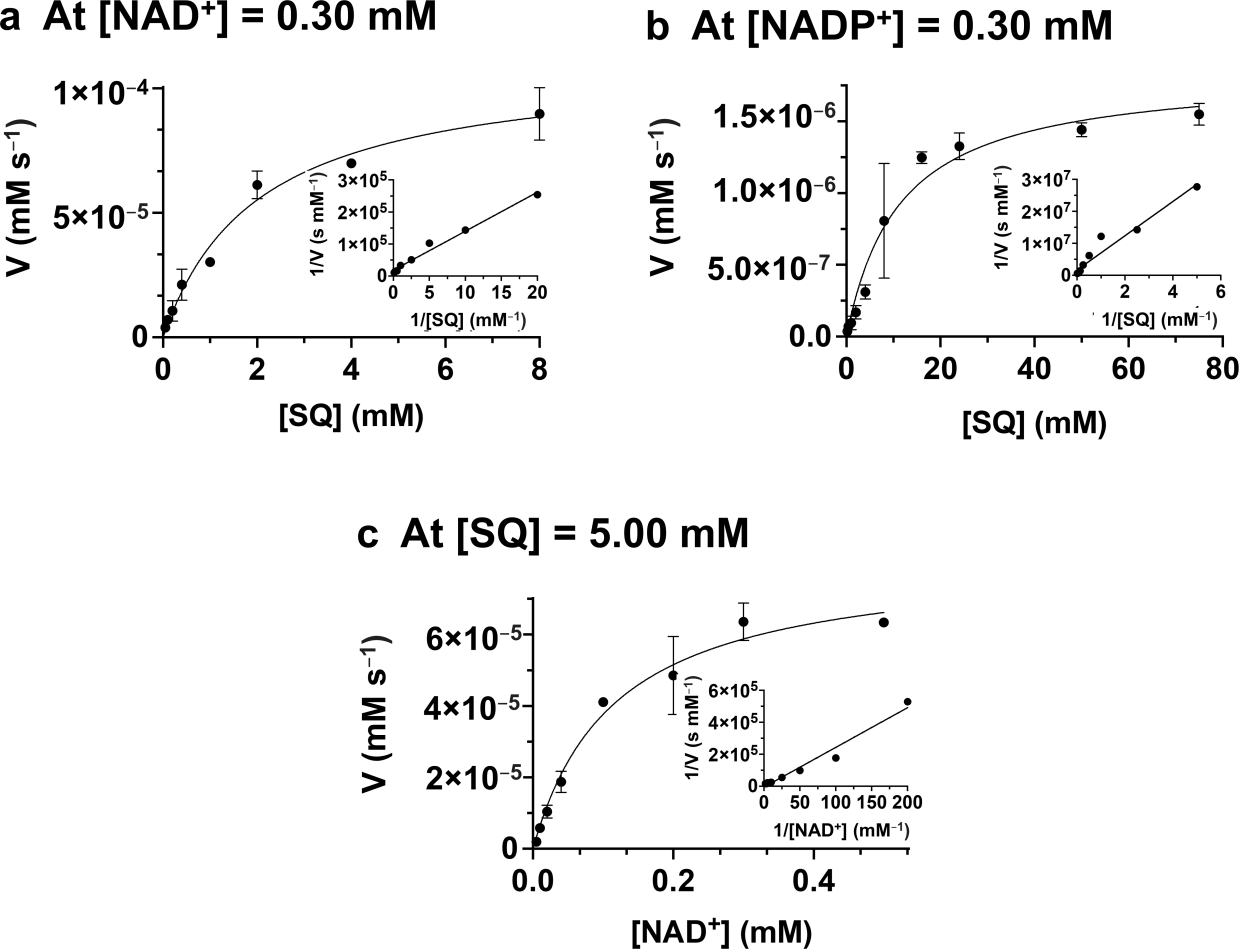
Pseudo-first-order kinetics for* Pp*SQDH. Kinetic studies and double reciprocal plots for (**a**) varying SQ at constant [NAD^+^] = 0.30 mM; (**b**) varying SQ at constant [NADP^+^] = 0.30 mM; (**c**) varying NAD^+^ at constant [SQ] = 5.0 mM. Reactions were conducted in 50 mM tricine buffer (pH = 8.5) with 1.4 nM SQ dehydrogenase. All plots show the mean of reaction rates (performed in triplicate). Error bars show standard error mean.

We synthesized a gene PpSQ1_0090A encoding *P. putida* SQ dehydrogenase (*Pp*SQDH) that was codon harmonized for expression in *Escherichia coli. Pp*SQDH was expressed with an N-terminal His_6_-tag and was purified using standard immobilized metal affinity chromatography (Supplementary Figure S1). Using Bis-Tris-Propane, the enzyme exhibited activity that climbed at higher pH values to the buffer limit of pH 9.5 (Supplementary Figure S2). However, the enzyme was more stable in tricine buffer, which has a practical buffering range of up to pH 8.5, and thus this condition was selected for subsequent work. Previously, recombinant *Pp*SQDH was reported to have approximately 100-fold preference for NAD^+^ versus NADP^+^; however, the details of how this analysis was performed are incomplete [10]. We, therefore, repeated this work by collecting kinetic parameters for SQ, NAD^+^, and NADP^+^ under pseudo-steady-state conditions in which the concentration of one substrate was varied while the other was held constant (Figure 2). Initially, we measured kinetic parameters for SQ under conditions in which NAD(P)^+^ was held constant at 0.3 mM. Under these conditions, SQ displayed a fivefold lower *K*_M_^app^ value, and a million-fold higher *k*_cat_^app^ value when using NAD^+^ versus NADP^+^ as a cofactor (Table 1). This corresponds to a million-fold preference for NAD^+^ in terms of (*k*_cat_/*K*_M_)^app^ values. With the cofactor preference for *Pp*SQDH clearly established as NAD^+^, we then measured pseudo-steady-state kinetics for NAD^+^ at a constant concentration of SQ (5 mM), affording *k*_cat_^app^ = 58 s^−1^, *K*_M_^app^ = 0.12 ± 0.05 mM, and (*k*_cat_/*K*_M_)^app^ = 500 mM^−1^ s^−1^.

**Table 1:** Kinetic parameters determined from recombinant *Pp*SQDH (PpSQ1_0090).

Variable substrate	Constant substrate	*k*_cat_ (s^−1^)	*K*_M_^app^ (mM)	*k*_cat_/*K*_M_^app^ (mM^−1^ s^−1^)
SQ^1^	NAD^+^ (0.30 mM)	79 ± 8.5	2.0 ± 0.7	39 ± 12
SQ^1^	NADP^+^ (0.30 mM)	(5.4 ± 0.7) x 10^−5^	11 ± 4.4	(4.8 ± 0.2) x 10^−5^
NAD^+1^	SQ (5.0 mM)	58 ± 7.6	0.12 ± 0.05	500 ± 150
G6P^1^	NAD^+^ (0.30 mM)	–	–	1.1 × 10^−7^

1 50 mM tricine buffer (pH 8.5).

We next examined whether *Pp*SQDH exhibits a preference for SQ versus the structurally analogous compound glucose-6-phosphate (G6P), which has previously been reported to not be a substrate [10]. By conducting the enzyme assays at high enzyme concentration, we could detect a low rate of reaction in the presence of the preferred cofactor, NAD^+^, at 0.3 mM. The plot did not exhibit saturation, so individual *k*_cat_^app^ and *K*_M_^app^ values could not be determined (Supplementary Figure S3). However, this allowed calculation of (*k*_cat_/*K*_M_)^app^ = 1.1 x 10^−7^ mM^−1^ s^−1^. Comparison of the (*k*_cat_/*K*_M_)^app^ values for SQ and G6P at [NAD^+^] = 0.3 mM reveals an estimated 10^8^ preference for the sulfosugar.

**Figure 3:**
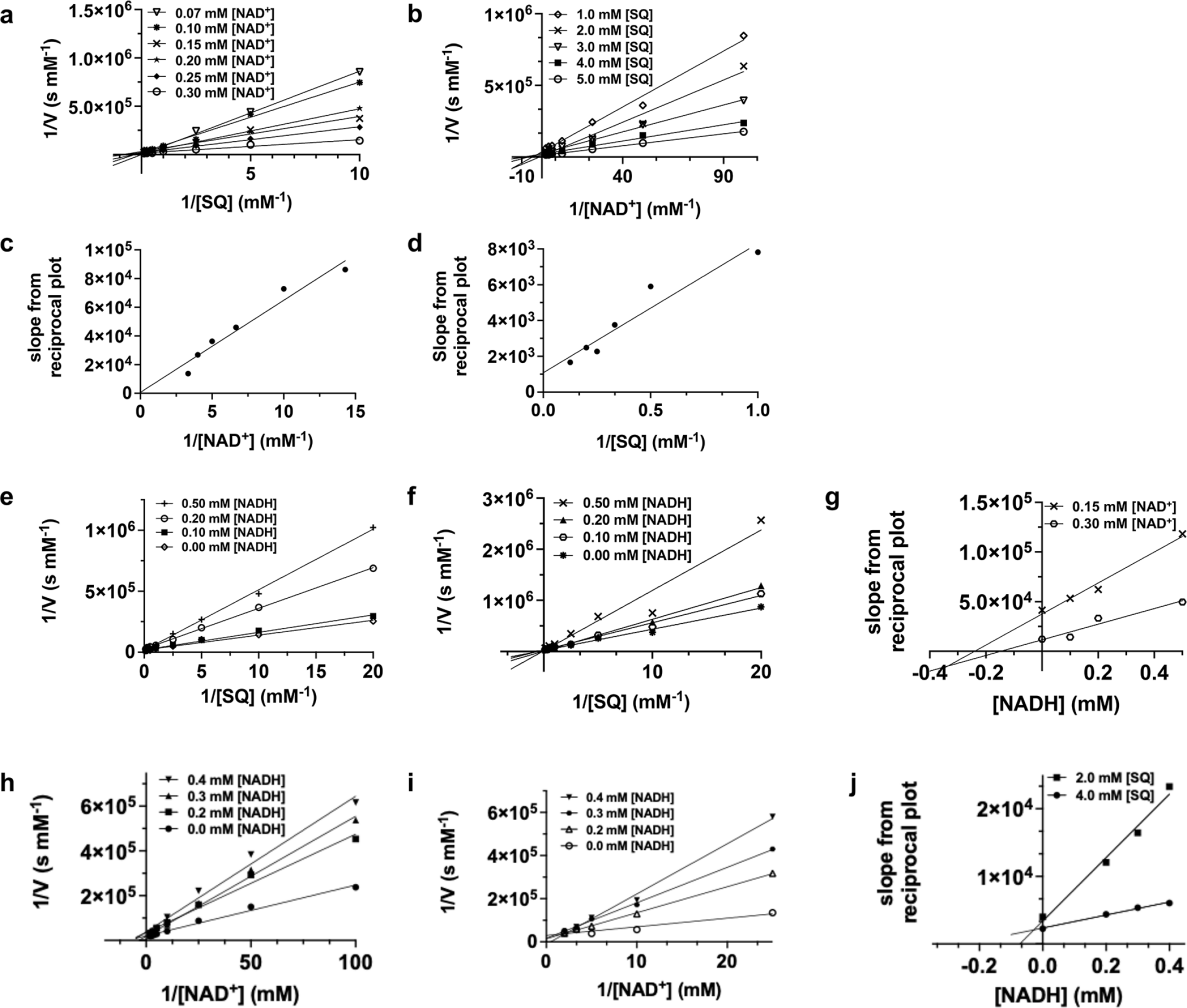
*Pp*SQDH uses a rapid equilibrium sequential mechanism. Bisubstrate kinetics: Ddouble reciprocal primary plots of (**a**) varying SQ concentrations at several fixed concentrations of NAD^+^ (0.07–0.30 mM), and (**b**) varying NAD^+^ concentrations at several fixed concentrations of SQ (1.00–5.00 mM). Secondary plots of slopes from primary double reciprocal plots for (**c**) varying concentrations of NAD^+^ (0.07–0.30 mM) and (**d**) varying concentrations of SQ (1.00–5.00 mM). Product inhibition studies: Ddouble reciprocal primary plots of varying [SQ] at several fixed concentrations of NADH (0–0.50 mM) at (**e**) 0.30 mM NAD^+^, and (**f**) 0.15 mM NAD^+^. (**g**) Secondary replot of slopes from reciprocal plots in (**e**) and (**f**), versus [NADH]. Double reciprocal primary plots of varying [NAD^+^] at several fixed concentrations of NADH (0–0.40 mM) at (**h**) 4.0 mM SQ, and (**i**) 2.0 mM SQ. (**j**) Secondary replot of slopes from reciprocal plots in (**h**) and (**i**), versus [NADH]. Reactions were conducted in 50 mM tricine buffer (pH = 8.50) using 1.4 nM SQ dehydrogenase. All data show the mean of reaction rates (performed in triplicate).

*Pp*SQDH uses two substrates and produces two products, and thus, its molecularity is referred to as bi-bi. Such enzymes react through a range of kinetic mechanisms that include rapid equilibrium in which both substrates bind to generate a ternary complex before a chemical step occurs or ping-pong in which one product is released prior to the binding of the second substrate. We investigated the kinetic mechanism of *Pp*SQDH using bisubstrate kinetic analysis [22]. We measured a series of Michaelis-Menten curves in which the concentration of NAD^+^ was varied, at various fixed concentrations of SQ. A double reciprocal plot of velocity against the concentration of NAD^+^ at the fixed concentrations of SQ gives a family of lines that intersect at a common point (Figure 3a). Similarly, a double reciprocal plot of velocity against the concentration of SQ at various fixed concentrations of NAD^+^ also gives a family of lines that intersect at a common point (Figure 3b). The interpretation of this graphical solution rules out the ping-pong mechanism that requires the dissociation of one product before the addition of the second substrate and instead exhibits a series of parallel lines. Secondary plots of the slopes of the double reciprocal plots gave a linear correlation, with the plot of the slopes derived from the SQ kinetic data passing through the origin, while the plot of slopes derived from the NAD^+^ kinetic data did not (Figure 3c and d). These data are consistent with a rapid equilibrium ordered binding mechanism in which NAD^+^ binds first [22], which is a typical feature of many bimolecular NAD(P)^+^-dependent oxidoreductases.

Product inhibition studies can be useful to establish the order of substrate binding [22]. NADH was examined as an inhibitor of the enzyme-catalyzed reaction of SQ and NAD^+^. Double reciprocal replots at two different [NAD^+^] showed intersection on the *y*-axis when [SQ] was varied, consistent with NADH acting as a competitive inhibitor for NAD^+^. A secondary replot of the slopes from the double reciprocal plots versus 1/[NADH] gave a pair of lines that intersected close to the *x*-axis. Likewise, double reciprocal replots of rates at two different [SQ] as a function of [NAD^+^] also gave a series of lines that intersected close to the *y*-axis. The secondary plot of the slopes from the double reciprocal plots versus 1/[NADH] gave two lines that intersected above the *x*-axis. These data are consistent with the bisubstrate kinetic analysis, namely a rapid equilibrium ordered binding mechanism in which NAD^+^ binds first, and suggest that NADH is the final product released.

### Structural analysis of SQ dehydrogenase

**Figure 4:**
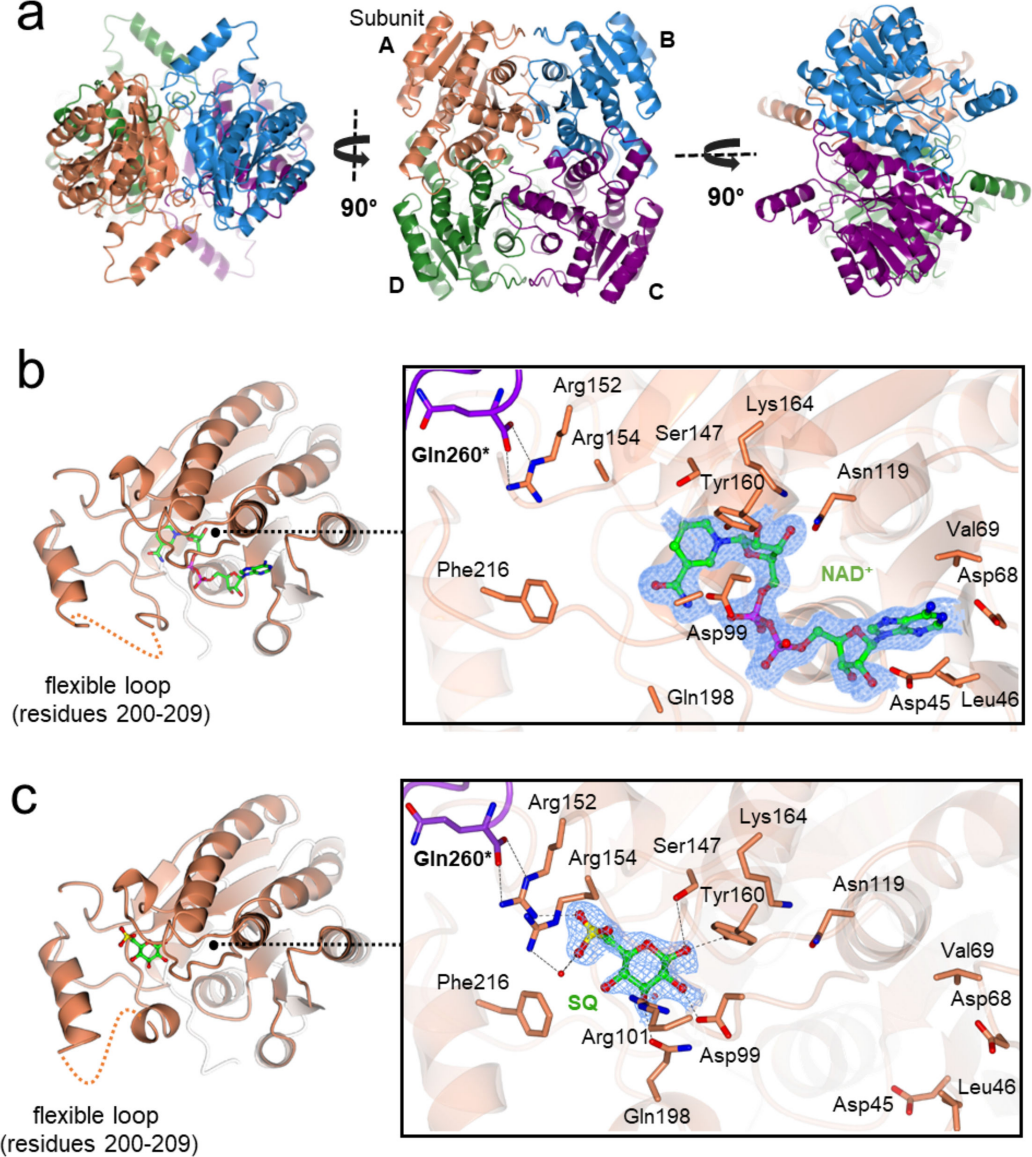
3D structures of apo-*Pp*SQDH and complexes with NAD^+^ or SQ. (**a**) Orthogonal views showing quaternary tetrameric assembly of apo-SQDH in cartoon representation. (**b**) SQDH monomer (left, in coral) and close -up view of SQDH•NAD^+^ complex (right) showing cofactor binding pocket. (**c**) SQDH monomer with flexible substrate loop (left, in coral) and close -up view of the active site of SQDH showing SQ -binding interactions. Residues lining the binding sites within 4 Å vicinity of the ligands are shown in cylinder format. Electron density in blue mesh corresponds to 2Fo − Fc map contoured at 0.7σ (0.196 e/Å^3^).

To elucidate the enzyme-substrate interactions of *Pp*SQDH, 3D X-ray crystal structures of apo-*Pp*SQDH as well as binary complexes of *Pp*SQDH•NAD^+^ and *Pp*SQDH•SQ were obtained to resolutions of 1.7, 1.9 and 1.9 Å, respectively, each in the I2 space group with two molecules in the asymmetric unit. Size exclusion chromatography-multi angle laser light scattering (SEC-MALLS) analysis detected a dominant solution state species of ~119 kDa (monomer molecular weight of 28,000 Da, confirmed by LC-MS analysis), thereby demonstrating a tetrameric assembly of *Pp*SQDH in solution as commonly seen for members of the SDR family (Supplementary Figure S1). PISA (Proteins Interfaces, Surface & Assemblies) analysis [23] shows that the compact tetrameric assembly of *Pp*SQDH subunits buries a total of 13,974 Å^2^ of solvent-accessible surface area, which accounts for 29% of the total surface area of the oligomer (Figure 4a).

*Pp*SQDH adopts the classical SDR fold with a highly conserved active site architecture comprising the Asn119-Ser147-Tyr160-Lys164 catalytic tetrad [24,26]. Each subunit consists of an N-terminal Rossmann fold with a central, parallel β-sheet (β1–7) sandwiched by α helices on either side, followed by a smaller C-terminal region. Pairwise distance matrix-alignment (DALI) analysis [27] showed close structural homologs from the SDR superfamily with diverse substrate specificities such as 3-oxo-[acyl-carrier-protein] reductase from *Rhizobium meliloti* (PDB ID: 4DRY with a DALI Z score of 30.1, 2.1 Å root mean square deviation (rmsd) over 229 residues and 26% sequence ID) and biphenyl dehydrogenase from *Pandoraea pnomenusa* str. B-356 [28] (PDB ID: 3ZV3 with a Z score of 30.7, 2.0 Å rmsd over 239 residues, and 31% sequence ID) (Supplementary Figure S5). Of interest, two bacterial dehydrogenases active on C2/C3 sulfonate substrates were identified amongst the hits: DHPS dehydrogenase HpsO from *Ruegeria pomeroyi* DSS-3 [29] (PDB ID: 8WWF, Z score of 28.3, 2.3 Å rmsd over 235 residues, and 26% sequence ID) and sulfoacetaldehyde reductase IsfD from *Klebsiella oxytoca* [30] (PDB ID: 6IXJ, Z score of 33.4, 1.9 Å rmsd/ 246 residues, and 29% sequence ID). These members of the SDR superfamily feature a characteristic, flexible substrate-binding loop at the mouth of the active site presumably for shielding the catalytic center from the solvent during reaction. We observe an ordered region with a higher B-factor (residues 195–218) in the crystal structure of ligand-free *Pp*SQDH close to the C-terminal end, which corresponds to the extensively documented substrate-binding loop of SDR dehydrogenases.

Crystallization of *Pp*SQDH in the presence of NAD^+^ allowed determination of the 3D structure of the *Pp*SQDH•NAD^+^ binary complex (Figure 4b). Dinucleotide binding involves interactions with the Rossmann fold domain, including a hydrophobic pocket to accommodate the adenosine ribosyl group and a polar ribose binding residue (Asp45). The Rossmann fold contains the glycine-rich sequence GGASGIG that interacts with the nucleotide diphosphate group, and which is representative of the Gly-X_3_-Gly-X-Gly motif conserved in many SDR enzymes [31]. Unlike the large domain movements of the functionally related medium-chain and long-chain dehydrogenases upon NAD(P)(H) binding [31,32], the structure of the binary complex superposes with the ligand-free *Pp*SQDH structure with an rmsd of 0.2 Å across the entire protein sequence, with only localized changes of side-chain conformations involved in binding the nucleotide, and some minor movements of substrate-binding loop residues. Binding of the preferred cofactor, NAD(H), involves H-bonds with 2′-OH and 3′-OH of the adenosine ribosyl moiety and Asp45. This residue constitutes the well-described acidic residue that confers NAD(H) specificity for SDR enzymes; NADP(H) preferring enzymes have instead two basic residues (Arg or Lys) that bind to the 2′-phosphate [25]. Consistent with the structural observations, thermal shift assay using nanoscale differential scanning fluorimetry (nano-DSF) data gave a melting temperature (Tm) increase of +1°C in the presence of NAD^+^, while the Tm decreased by 4°C in the presence of NADP^+^ (Supplementary Figure S4). The thermal destabilization of enzyme may arise due to poor accommodation of the 2′-phosphate group of NADP^+^. At the active site, the *Pp*SQDH•NAD^+^ binary complex displays a conserved catalytic tetrad with the characteristic Tyr-X_3_-Lys motif [26], with Tyr160 and Lys164 H-bonding to the 2′- and 3′-hydroxyls of nicotinamide riboside, thereby projecting the reactive C4 center of the nicotinamide ring near the substrate binding pocket for catalysis.

The binary *Pp*SQDH•SQ complex was obtained by directly soaking SQ onto apo *Pp*SQDH crystals. Clear density in the omit map in one of the subunits allowed for modeling of SQ bound at the active site at an occupancy of 0.5 (Figure 4c). The sulfo-sugar makes several interactions with the active site residues: C1-OH makes H-bonding interactions with catalytic residues Tyr160 (2.7 Å) and Ser147 (3.2 Å); C2-OH is H-bonded to side-chain Asp99 (2.5 Å) and C3-OH with Gln198 (2.6 Å) and Arg101 (2.7 Å). The sulfonate pocket is well defined: the sulfonate oxyanion makes a salt bridge to Nɳ1 Arg152 (2.8 Å) while another sulfonate oxygen is hydrogen bonded to a water molecule at a distance of 2.6 Å. In turn, this water molecule makes H-bonding interactions with Arg154 (3.1 Å) and another water molecule. Binding of SQ is associated with disorder in the substrate-binding loop such that the electron density of residues 201–207 became uninterpretable compared to the ligand-free structure, indicating a possible role for this region in substrate binding and catalysis.

**Figure 5:**
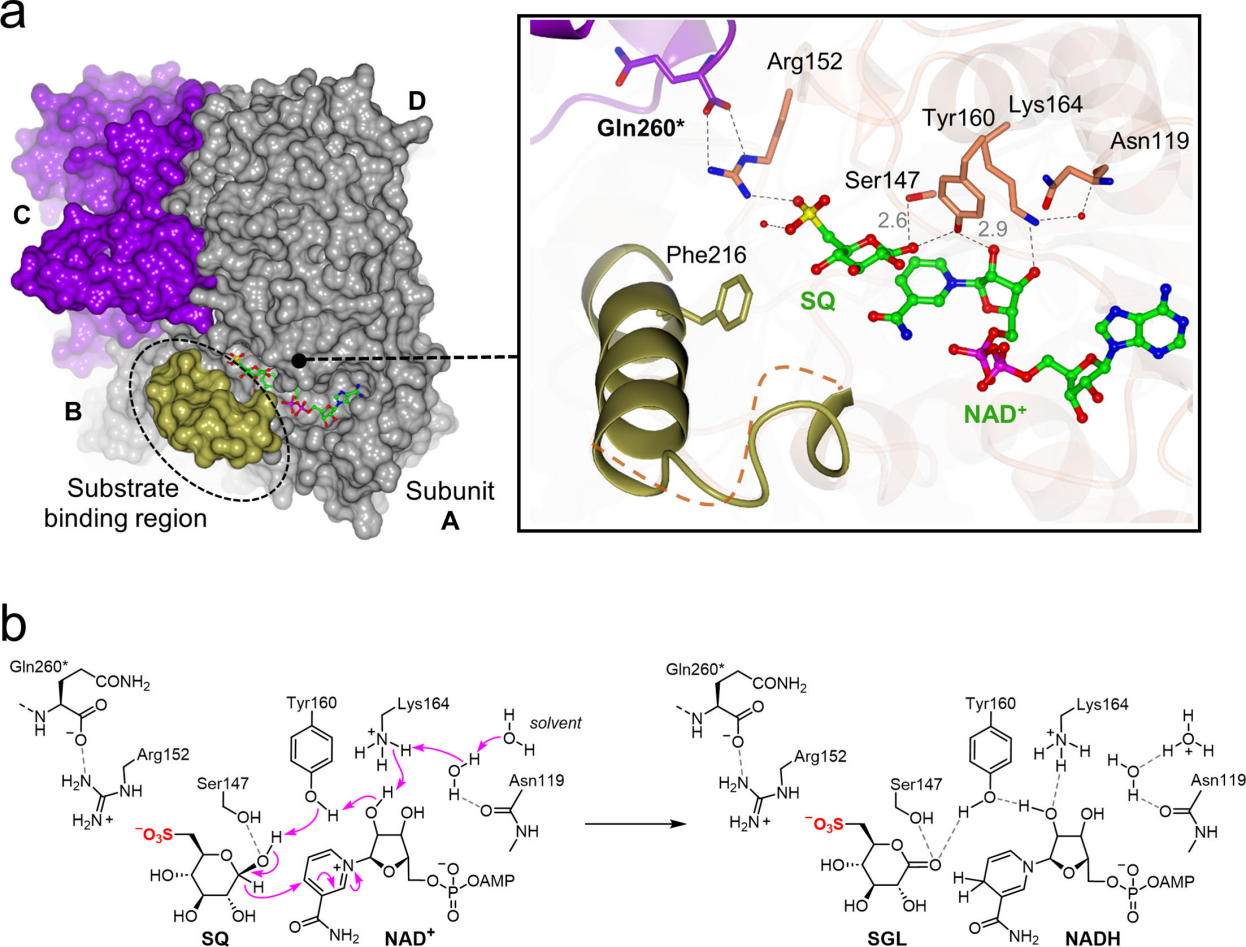
Visualization of a ternary *Pp*SQDH•NAD^+^•SQ complex, conformational changes upon substrate binding, and proposed reaction mechanism. (**a**) Surface representation of tetrameric apo-SQDH structure with interpretable density of binding loop (in burnt yellow) that closes over the substrate binding pocket; the diagonal subunit C that makes C-terminal tail interactions with the substrate binding site is shown in purple (left). Overlay of apo-SQDH, SQDH•NAD^+^ and SQDH•SQ structures (right, inset) allow visualization of conformational change binding events for SQDH: the flexible substrate -binding loop, relative location and orientations of co-substrate binding pockets. The C1 atom of SQ is within 3 Å distance of C4 of Thethe cofactor. The flexible loop in apo-SQDH (greay) becomes disordered in the SQDH•SQ structure,, suggesting a role in binding SQ. (**b**) Proposed reaction mechanism for *Pp*SQDH.

Attempts to trap the ternary complex by soaking crystals of the *Pp*SQDH•NAD^+^ binary complex with SQ were unsuccessful, giving either poorly diffracting crystals or the resulting structure did not show clear density for SQ and was associated with disorder in the substrate-binding loop. Instead, overlay of the structures of the *Pp*SQDH•NAD^+^ and *Pp*SQDH•SQ binary complexes provided a model of the *Pp*SQDH•NAD^+^•SQ ternary complex. In this model, the nicotinamide C4 atom is within 3 Å of the SQ C1-OH, which in turn is H-bonded to the proposed catalytic Tyr160 residue, poised for hydride transfer from C1 of SQ to C4 of the nicotinamide ring (Figure 5).

### Structural comparison of SQ dehydrogenase with other alkylsulfonate dehydrogenases

**Figure 6:**
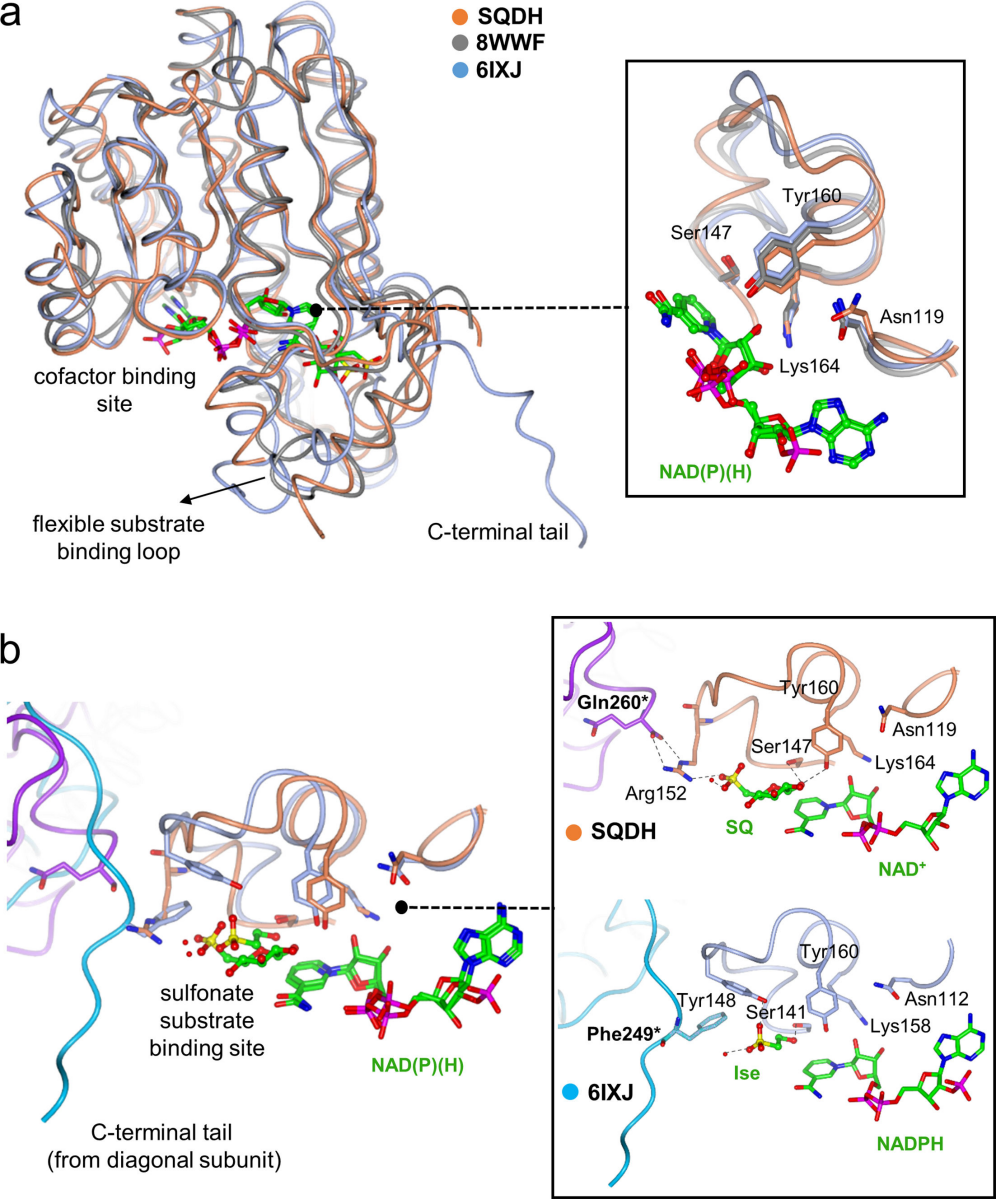
Superposition of 3D structures of SDR members that recognize sulfonate substrates. (**a**) An overlay of structures of subunit A of *Pp*SQDH (coral) with sulfoacetaldehyde reductase from *K. oxytoca* (6IXJ.pdb; ice blue), *R*-DHPS dehydrogenase HpsO from *R. pomeroyi* (8WWF.pdb; greay) to show the overall fold similarities consisting of Rossmann fold for nucleotide binding and the catalytic tetrad Tyr-Ser-Lys-Asn residues (*Pp*SQDH numbering) are shown in the inset. (**b**) Close -up view of the cofactor binding pockets in *Pp*SQDH versus sulfoacetaldehyde reductase (6IXJ.pdb) highlighting differences in the length of C-terminal tail and its interaction in sulfonate substrate binding site.

A range of biochemical pathways involving catabolism of short-chain C2- and C3-sulfonates have recruited SDR members that have evolved to recognize the sulfonate moiety. For example, *R. pomeroyi* HpsO is an NAD(P)H-dependent DHPS dehydrogenase found in DHPS catabolism pathways [17,29] and *K. oxytoca* IsfD is an NADPH-dependent sulfoacetaldehyde reductase from a taurine assimilatory pathway that differs from SQDH in cofactor preference [30]. Overlay of the structures of these three organosulfonate processing enzymes reveals spatially conserved arrangements of the Asn119-Ser147-Tyr160-Lys164 catalytic tetrad (Figure 6a). A comparison of the substrate-bound structures of *Pp*SQDH and *K. oxytoca* IsfD shows the reactive hydroxyl group in each substrate with similar locations relative to the catalytic tetrad. The 3D structure of IsfD in complex with isethionate and NADPH shows that the sulfonate group is recognized by Tyr148 and Gln244, via a bridging water molecule, and does not involve a basic residue equivalent to Arg152 in the *Pp*SQDH complex with SQ. The 3D structure of IsfD revealed a long C-terminal tail that participates in a cross-monomer interaction (from the diagonal subunit), resulting in the projection of Phe249* into the sulfonate substrate binding site [30], whereas the cross-monomer interaction in *Pp*SQDH involves a shorter tail such that Gln260* instead projects into a ‘second sphere’ interaction with the sulfonate binding residue Arg152 (Figure 6b).

## Discussion

The sulfo-ED pathway in *P. putida* catalyzes the partial degradation of SQ, yielding pyruvate, SL, and two NADH molecules [10]. *Pp*SQDH, the first enzyme in the pathway, oxidizes SQ to SGL, producing one NADH molecule. The second NADH is generated in the final step when SLA is oxidized to SL. *Pp*SQDH exhibits high specificity for NAD^+^ over NADP^+^. It belongs to protein family PF13561 (enoyl-acyl carrier protein reductase), which is part of the SDR superfamily (Interpro IPR002347). While the reaction catalyzed by SQDH resembles that of G6PDH, G6PDH enzymes belong to distinct PFAM families (PF00479, PF02781), have different folds, and exhibit a preference for NADP^+^ as a cofactor. The preference of *Pp*SQDH for NAD^+^ is aligned with the general preference for this cofactor in catabolic pathways such as glycolysis and the citric acid cycle, with the NADH produced used in oxidative phosphorylation. It also reflects the aerobic nature of *P. putida* in which SQ is used for respiratory energy metabolism [10].

Bisubstrate kinetic analysis provides evidence for a rapid equilibrium ordered mechanism for *Pp*SQDH in which NAD^+^ binds first, and NADH dissociates last, with the *Pp*SQDH•NAD^+^•SQ complex being the chemically reactive species that converts to *Pp*SQDH•NADH•SGL. Overlay of the 3D structures of the *Pp*SQDH•NAD^+^ and *Pp*SQDH•SQ binary complexes determined by X-ray crystallography allowed the development of a model of the *Pp*SQDH•NAD^+^•SQ ternary complex that enables the proposal of a mechanism for the enzyme-catalyzed reaction (Figure 5b). In this proposal, Tyr160 and Lys164 are the key catalytic residues that are conserved across the SDR superfamily. Accordingly, Lys164 serves as a general base to assist Tyr160 in deprotonating the anomeric hydroxyl, facilitating hydride transfer to C4 of the nicotinamide ring of NAD^+^. This proposed mechanism is consistent with that proposed for *Comamonas testosteroni* 3β/17β-hydroxysteroid dehydrogenase [26].

The SDR superfamily is a diverse group of enzymes with various activities, including oxidoreductases, hydrolases, lyases, ligases, and isomerases. Many SDR members are oxidoreductases that convert aldohexoses to sugar lactones. Examples include NADP^+^-dependent glucose 1-dehydrogenase, L-rhamnose 1-dehydrogenase, D-xylose dehydrogenase, *N*-acetyl-D-mannosamine dehydrogenase, and L-fucose dehydrogenase. However, to our knowledge, no SDR enzyme can oxidize 6-phosphoaldohexoses to the corresponding phosphoglyconolactones. Perhaps the closest chemically analogous reaction is catalyzed by glucitol-6-phosphate dehydrogenase (EC 1.1.1.140), which converts a 6-phosphoalditol to fructose-6-phosphate, a 6-phosphoaldoketose.

Several members of the SDR superfamily, specifically those within the PF13561 family, are oxidoreductases that act on organosulfonates. Examples include *R. pomeroyi* NADP^+^-dependent DHPS dehydrogenase HpsO [17,29], *K. oxytoca* NADPH-dependent sulfoacetaldehyde reductase IsfD (EC 1.1.1.313) [30], and *P. putida* SQ dehydrogenase, studied herein. Comparison of the 3D structures of these three organosulfonate-active enzymes reveals a high degree of structural similarity at the levels of the monomer and the quaternary structure of their tetramers. All three enzymes exhibit an interdomain interaction wherein the C-terminal tail of one monomer protrudes across into the diagonal monomer and forms part of the active site. However, the length of their C-terminal tails varies significantly. Sulfoacetaldehyde reductase IsfD possesses the longest tail, while DHPS dehydrogenase HpsO and SQ dehydrogenase *Pp*SQDH have much shorter tails. These cross-monomer interactions help provide a structural rationale for the formation of a tetramer in the solution and crystalline states.

## Conclusions

The present study elucidates the structural and kinetic features of *Pp*SQDH, the first pathway-specific enzyme in the sulfo-ED pathway of sulfoquinovose catabolism. Our work provides insight into how the versatile scaffold of SDR proteins has been adapted to allow recruitment into this sulfoglycolytic pathway. These findings contribute to a deeper understanding of how microorganisms participate in the biogeochemical sulfur cycle by processing organosulfur compounds. Our work complements broader efforts to characterize enzymes involved in the transformation of diverse organosulfur metabolites and to elucidate the structural basis of the recognition and processing of organosulfonates by their cognate catabolic enzymes.

## Methods

### Reagents

Reagents and buffers were purchased from Sigma-Aldrich and used as received. SQ was obtained from MGAT GmbH (Germany).

### Sequences

#### > PpSQ1_00090_coding_sequence

ATGGGCAGCAGCCATCATCATCATCATCACAGCAGCGGCATGAACCGTCATACTGATACCCACTACCCGTCTCTGGCTGACAAGGTAGTGCTGATTTCCGGTGGTGCTTCTGGCATTGGCCGTGCATTCGTTGAAGCCTTCGTAGCTCAGGGCTCTCGTGTGGCATTCCTGGATCTGGATGCAGAGGCAGGTCAGGGTCTGGCACACGCACTGGGTGCTAACTCCCTGTTCCTGCCGTGCGATGTGCGTGACATTGAACGTCTGAAAGCGTGTGTTGCCGAAGTTGAACGTACCTGGGGCGCGGTTGACGTTCTGATCAACAATGCAGCACGTGATGACCGTCACGCTCTGGCGGATGTTTCTGTCGAATACTGGGACGAACGCATGCAGACCAACCTGCGTCACGCATTTTTTGCAGCACAGGCAGTAGCACCGGGTATGGCTCGTCGTGGTTCTGGTGCGATTATCAACATGGGTAGCATCTCTTGGATGCGCGGCCGTCCAGGCATGGTATGCTATACCACTGCAAAAGCCGCTCTGAACGGCATGACCCGTACTCTGGCTCGCGAACTGGGTGGTCAGGGTATTCGTATCAACTCCCTGGTACCGGGTGCGATCCGTACTGAACGTCAGGATGCTATGTGGGCAGCTGACCCGGCTGGTCTGGAAGCAGCTTCCCAGGCTTTTATCGATCAACAGATGCTGAAATTCCGCCTGGACGCTTCTGATTGCGCTCGCCTGGCACTGTTTCTGGCCTCTGACGACTCTCGCGGCTGTACTGGCCAGAATTTCGTAGTTGACGCCGGCCTGTCTATCCAGTGA

#### > PpSQ1_00090 expressed protein sequence

MGSSHHHHHHSSGMNRHTDTHYPSLADKVVLISGGASGIGRAFVEAFVAQGSRVAFLDLDAEAGQGLAHALGANSLFLPCDVRDIERLKACVAEVERTWGAVDVLINNAARDDRHALADVSVEYWDERMQTNLRHAFFAAQAVAPGMARRGSGAIINMGSISWMRGRPGMVCYTTAKAALNGMTRTLARELGGQGIRINSLVPGAIRTERQDAMWAADPAGLEAASQAFIDQQMLKFRLDASDCARLALFLASDDSRGCTGQNFVVDAGLSIQ

### Cloning, expression, and purification of PpSQ1_00090 (*Pp*SQDH)

A gene encoding PpSQ1_00090 from *P. putida* SQ1 was codon harmonized for *E. coli*, synthesized, and cloned into the pET28 vector using the *NcoI* and *XhoI* restriction sites. This plasmid was transformed into chemically competent ‘NEB T7 Express’ *E. coli*, plated onto LB-agar (50 μg/ml kanamycin), and incubated at 37°C for 16 h. A single colony was used to inoculate 10 ml of LB media containing 50 μg/ml kanamycin, followed by incubation at 37°C for 16 h. This culture was used to inoculate 1000 ml of ‘S-broth’ (35 g tryptone, 20 g yeast extract, 5 g NaCl, pH 7.4) containing 50 μg/ml kanamycin, which was incubated with shaking (250 rpm) at 37°C until it reached an A_600_ of 0.7. The culture was cooled to room temperature, isopropyl 1-thio-β-D-galactopyranoside added to a final concentration of 400 μM, and then incubated with shaking (200 rpm) at 18°C for 16 h. The cells were harvested by centrifugation at 17,000 × *g* for 20 min at 4°C then resuspended in 40 ml of binding buffer (50 mM NaP_i_, 500 mM NaCl, 5 mM imidazole, pH 7.5) containing protease inhibitor (Roche complete EDTA-free protease inhibitor cocktail) and lysozyme (0.1 mg ml^-1^) by nutating at 4°C for 30 min. Benzonase (1 μl) was added to the mixture and lysis was effected by sonication. The lysate was clarified by centrifugation (17,000 × *g* for 20 min at 4°C), the supernatant filtered (0.45 μm), and then loaded onto a 1 ml HisTrap HP column (GE Healthcare). The column was washed with 15 ml binding buffer and the protein was eluted using elution buffer (50 mM NaP_i_, 500 mM NaCl, 500 mM imidazole, pH 7.5). Fractions containing the protein of interest (as determined by SDS-PAGE) were further purified by size exclusion chromatography on a Superdex 75 10/300 GL column using 50 mM NaP_i_, 150 mM NaCl, pH 7.5.

For the preparation of crystallization samples, the plasmids containing the *PpSQDH* gene were used to transform *E. coli* BL21(DE3) competent cells for expression. Starter cultures were grown in LB medium (5 ml) containing 30 µg ml^−1^ kanamycin for 18 h at 37°C with shaking at 220 rpm. One liter volume cultures were inoculated with the starter culture (5 ml) and incubated at 37°C with shaking at 220 rpm until an OD600 of 0.6–0.8 was reached. Gene expression was induced by the addition of isopropyl 1-thio-β-D-galactopyranoside (0.5–1 mM) and shaking was continued overnight at 18°C at 200 rpm. The cells were then harvested by centrifugation at 5000 × *g* for 20 min and resuspended in 50 mM Tris buffer pH 7.5, containing 300 mM NaCl and 30 mM imidazole. Cells were disrupted using a high-pressure cell homogenizer at 20 k psi, and the suspension was centrifuged at 50,000 × *g* for 30 min to yield a clear lysate. The N-terminal His6-tagged protein was purified using immobilized-metal affinity chromatography (IMAC) using nickel nitrilotriacetic acid (Ni-NTA) column, followed by size exclusion chromatography (SEC) (Supplementary Figure S1). For IMAC, the lysate was loaded onto a pre-equilibrated Ni-NTA column, followed by washing with a load buffer (50 mM Tris, 300 mM NaCl, 30 mM imidazole pH 7.5). The bound protein was eluted using a linear gradient with buffer containing 300 mM imidazole. Protein fractions were pooled, concentrated, and loaded onto a HiLoad 16/600 Superdex 75 gel filtration column pre-equilibrated with 50 mM Tris, 300 mM NaCl, pH 7.5 buffer. The protein was concentrated using a Vivaspin® centrifugal concentration with a 10 kDa molecular weight cut-off to a final concentration of 20–40 mg/ml for crystallization experiments.

### Protein characterization

#### SEC-MALLS analysis

Experiments were conducted on a system comprising a Wyatt HELEOS-II multi-angle light scattering detector, and a Wyatt rEX refractive index detector linked to a Shimadzu HPLC system (SPD-20A UV detector, LC20-AD isocratic pump system, DGU-20A3 degasser and SIL-20A autosampler). Work was conducted at room temperature (20 ±2°C). The solvent was 0.2 µm filtered before use and a further 0.1 µm filter was present in the flow path. The column was equilibrated with at least two column volumes of solvent before use, and flow was continued at the working flow rate until baselines for UV (280 nm), light scattering, and refractive index detectors were all stable. The sample injection volume was 100 µl at ~2 mg/ml concentration; Shimadzu LabSolutions software was used to control the HPLC and Astra 7 software for the HELEOS-II and rEX detectors. The Astra data collection was 1 minute shorter than the LC solutions run to maintain synchronization. Blank buffer injections were used as appropriate to check for carry-over between sample runs. Data were analyzed using the Astra 7 software. MWs were estimated using the Zimm fit method with degree 1. A value of 0.182 was used for protein refractive index increment (dn/dc). The running buffer was 100 mM Tris pH 7.5, 300 mM NaCl, which was used to equilibrate the Superdex S200-2 10/300 GL #10,245,604 column (GE Healthcare), and experiments were conducted at a flow rate of 0.5 ml/min with a sample injection volume (nominal injection mass of ~100–200 µg) of 100 µl at 20 °C.

#### Protein mass spectrometry analysis

A 2 µl sample of target protein at 0.5 mg/ml was diluted up to 100 µl in 0.1% TFA. The protein sample was then desalted using an MSPac DS-10 Desalting Cartridge (Thermo Scientific) prior to infusion at 0.2 ml/min into a Bruker maXis-HD qTOF mass spectrometer. Positive ESI-MS spectra were continuously acquired over a 5-min chromatography gradient. Multiply charged species were deconvoluted to average neutral masses using maximum entropy deconvolution, followed by Apex peak picking (S/*N* > 10). Instrument control, data acquisition, and processing were performed using Compass 1.7 software (microTOF control and DataAnalysis, Bruker Daltonics).

#### Nanoscale DSF analysis

NanoDSF studies were performed on a Prometheus NT.48 (NanoTemper). Data recording and initial analysis were performed with PR.ThermControl software. SQDH samples were at 1 mg/ml in 50 mM Tris, 300 mM NaCl pH 7.5, and pre-incubated with 5 mM ligand at 25°C. All samples were centrifuged at 13,000 rpm for 2 min prior to loading, and 15 μl was loaded onto the capillary per sample. Experiments were performed in duplicate with the temperature ramp from 25°C to 95°C, at 1.0°C/min with 20% excitation power.

### Enzyme kinetics

#### General

Phosphate buffer was not used in our investigations of *Pp*SQDH as it has been shown that NADH and NADPH are unstable in phosphate buffer [33]. Sulfonate buffers were also avoided due to possible interactions within the sulfonate binding pocket of *Pp*SQDH. Instead, we opted to employ the Good’s buffer, tricine. The production of NAD(P)H from oxidation of NAD(P)^+^ was monitored using a UV-visible spectrophotometer at 340 nm. The extinction coefficient used for NADH and NADPH was 6363 M^–1^ cm^–1^.

#### pH profile for PpSQDH

Reactions were conducted at 20°C and contained 50 mM tricine buffer (at varying pH), [NaCl] = 150 mM, [NAD^+^] = 0.3 mM. Reactions were conducted at [SQ] = 0.1 mM (approximately *K*_M_/5) and initiated by addition of enzyme to a final concentration of 1.4 nM. The apparent kinetic parameters, *k*_cat_, *K*_M_, and (*k*_cat_*/K*_M_), were calculated using the Prism 9 software package (GraphPad Scientific Software). *k*_cat_*/K*_M_ was plotted against pH using a non-linear regression (curve-fit) to a third-order polynomial. Error bars show standard error mean. The highest reaction rate was at pH 9.0. However, we opted to use pH 8.5 due to a higher stability for the enzyme at this pH, which was used for all further kinetic work.

#### Kinetic analysis of PpSQDH

Kinetic analysis was performed for SQ, glucose-6-phosphate (G6P), NAD^+^, and NADP^+^ under pseudo first-order conditions in which the concentration of one substrate was varied while the other was held constant and *vice versa*. All reactions were performed in triplicate in 50 mM tricine buffer (at pH 8.5), with [NaCl] = 150 mM. The apparent kinetic parameters, *k*_cat_, *K*_M_, and (*k*_cat_*/K*_M_), were calculated using the Prism 9 software package (GraphPad Scientific Software). Error bars show standard error mean.

For variable [SQ] with constant [NAD^+^], reactions were conducted with varying concentrations of SQ (0.10–8.0 mM), constant [NAD^+^] = 0.30 mM, and [*Pp*SQDH] = 1.4 nM.
For variable [SQ] with constant [NADP^+^], reactions were conducted with varying concentrations of SQ (0.20–75 mM), constant [NADP^+^] = 0.30 mM, and [*Pp*SQDH] = 1.4 nM.
For variable [NAD^+^] with constant [SQ], reactions were conducted with varying concentrations of NAD^+^ (0.005–0.5 mM), constant [SQ] = 5.0 mM, and [*Pp*SQDH] = 1.4 nM.
For variable [G6P] with constant [NAD^+^], reactions were conducted with varying concentrations of G6P (16–200 mM), constant [NAD^+^] = 0.30 mM, and [*Pp*SQDH] = 0.48 mM.

#### Bisubstrate kinetic analysis

Reactions were performed in triplicate in 50 mM tricine buffer (at pH = 8.5), [NaCl] = 150 mM, [*Pp*SQDH] = 1.4 nM. A series of Michaelis–Menten plots were generated under pseudo first-order conditions in which the concentration of one substrate (either SQ or NAD^+^) was varied while the other was held constant for each plot. Double reciprocal ‘primary’ plots of each set of Michaelis–Menten data were generated, and the slopes from each were plotted versus 1/[SQ] or 1/[NAD^+^] to give ‘secondary plots’. All plots were generated using Prism 9 software package (GraphPad Scientific Software). Error bars show standard error mean.

For variable SQ concentration, [SQ] was varied over the range of 0.10–8.0 mM at six fixed concentrations of NAD^+^ (0.07−0.30 mM).
For variable NAD^+^ concentration, [NAD^+^] was varied over the range of 0.07−–0.30 mM at five fixed concentrations of SQ (1.0–5.0 mM).

#### Product inhibition studies

Product inhibition studies for *Pp*SQDH were conducted using the product NADH as the inhibitor. Reactions were performed in triplicate in 50 mM tricine buffer (at pH = 8.5), [NaCl] = 150 mM, [*Pp*SQDH] = 1.4 nM. A series of Michaelis–Menten plots were generated by measuring rates under pseudo-first-order conditions in which the concentration of one substrate was varied while the other substrate and the inhibitor NADH were held constant for each plot. The set of Michaelis–Menten plots acquired at two fixed concentrations of SQ or NAD^+^, and four concentrations of NADH, were used to construct double reciprocal ‘primary plots’. ‘Secondary plots’ were constructed for the slopes from the double reciprocal plots versus [NADH]. All plots were generated using Prism 9 software package (GraphPad Scientific Software). Error bars show standard error mean.

At [NAD^+^] = 0.30 mM, reactions were conducted with varying concentrations of SQ (0.10–8.0 mM) and four fixed concentrations of NADH (0.00−0.50 mM).
At [NAD^+^] = 0.15 mM, reactions were conducted with varying concentrations of SQ (0.05–8.0 mM) and four fixed concentrations of NADH (0.00−0.50 mM).
At [SQ] = 4.0 mM, reactions were conducted with varying concentrations of NAD^+^ (0.04−0.30 mM) and four fixed concentrations of NADH (0.00−0.40 mM).
At [SQ] = 2.0 mM, reactions were conducted with varying concentrations of NAD^+^ (0.04−0.30 mM) and four fixed concentrations of NADH (0.00−0.40 mM).

### Initial crystallization screening and optimized crystallization conditions

Initial screening was performed using commercially available INDEX, Crystal HT, PEG/ion HT™ (from Hampton Research) and PACT premier (Molecular Dimensions) screens in 96-well sitting drop trays. Further optimization was carried out in a 48-well sitting drop or 24-well hanging-drop format to obtain optimal crystals for X-ray diffraction.

A crystal of ligand-free apo-SQDH was grown using a 20 mg/ml protein solution in 50 mM TRIS buffer pH 7.5 containing 300 mM NaCl in a drop with 0.15 μl protein: 0.15 μl mother liquor, the latter comprising 0.1 M MIB (sodium malonate dibasic monohydrate, imidazole, boric acid) buffer pH 7.0 and 25% w/v PEG (polyethylene glycol) 1500. SQDH•NAD complex was obtained using co-crystallization of a protein solution at 20 mg/ml in 50 mM TRIS buffer, 300 mM NaCl buffer pH 7.5 used to set up a drop containing 0.1 μl protein: 0.15 μl mother liquor, the latter comprising 0.2 M magnesium chloride, 0.1 M HEPES buffer pH 7.5, 25% v/v polyethylene glycol 3350. Binary SQDH•SQ complex was obtained by soaking SQ on a crystal of apo-SQDH grown using a 20 mg/ml protein solution in 50 mM TRIS buffer pH 7.5 containing 300 mM NaCl in a drop with 0.15 μl protein: 0.15 μl mother liquor, the latter comprising 0.1 M magnesium formate and 15% w/v PEG 3350. Crystals were harvested into liquid nitrogen, using nylon CryoLoops^TM^ (Hampton Research) using mother liquor without any cryo-protectants. Data were collected at Diamond Light Source, Didcot, Oxfordshire, U.K., on beamline I03 under project MX32736.

### Data collection, processing, and refinement

The data were processed and integrated using XDS [34] and scaled using SCALA [35] included in the Xia2 processing system [36,37]. Data collection and refinement statistics are given in Supplementary Table S1. Structures of native and binary complexes were solved using MrBUMP [38] within the automated molecular replacement pipeline in CCP4-cloud suite [39], using AlphaFold structure [40,41] (AF-P0DOV5) as the initial search model. The structure was built and refined using iterative cycles using COOT [42] and REFMAC [43], the latter employing local NCS restraints. Following the building and refinement of the protein and water molecules, clear residual density was observed in the omit maps for metals and ligands. The coordinate and refinement library files for ligands were prepared using ACEDRG [44]. In the SQDH•NAD complex structure, NAD was modeled at an occupancy of 0.5 in each chain. In the SQDH•SQ binary complex, SQ was modeled at an occupancy of 0.5 in each chain A, and additional partial density seen at the active site in chain B was left unmodeled. All steps were performed from within the CCP4i/CCP4i2 suite [45,46]. The coordinate files and structure factors have been deposited in the Protein Data Bank with accession numbers: 9GWU (apo-SQDH), 9GWV (SQDH•NAD), and 9GWW (SQDH•SQ).

## Supplementary material

online supplementary material 1.

## Data Availability

X-ray structural data of *Pp*SQDH are available through the RCSB Protein Databank using accession codes 9GWU, 9GWV, and 9GWW. All other data are available in the manuscript and supporting information figures and tables.

## Abbreviations

Coot: Crystallographic Object-Oriented Toolkit
DHPS: 2,3-dihydroxypropanesulfonate
ED: Entner-Doudoroff
EMP: Embden-Meyerhof-Parnas
G6P: glucose-6-phosphate
KDSG: 2-keto-3-deoxysulfogluconate
NAD+: nicotinamide adenine dinucleotide
NCS: Non-crystallographic symmetry
PpSQDH: *P. putida* SQ dehydrogenase
REFMAC: REFinement of MACromolecular structures
SCALA: scale together multiple observations of reflections
SDR: short-chain dehydrogenase/reductase
SFT: sulfofructose transaldolase
SGL: sulfogluconolactone
SL: sulfolactate
SLA: sulfolactaldehyde
SQ: sulfoquinovose
SQDG: sulfoquinovosyl diacylglycerol
SQGro: sulfoquinovosyl glycerol
