# Supplementary material for: Structure, kinetics, and mechanism of Pseudomonas putida sulfoquinovose dehydrogenase, the first enzyme in the sulfoglycolytic Entner-Doudoroff pathway
Source: Biochem J. 2025 Jan 22;482(2):BCJ20240605. doi: 10.1042/BCJ20240605 (PMC12133307; doi:10.1042/BCJ20240605)
Supplement: online supplementary material 1. [file bcj-482-2-BCJ20240605-s001.docx]

**Structure, kinetics and mechanism of *Pseudomonas putida* sulfoquinovose dehydrogenase, the first enzyme in the sulfoglycolytic Entner-Doudoroff pathway**

Laura Burchill,^1†^ Mahima Sharma,^2†^ Niccolay Madiedo Soler,^3,4^ Ethan D. Goddard-Borger,^3,4^ Gideon J. Davies,^2^* Spencer J. Williams^1^*

**Supplementary Figures**

**
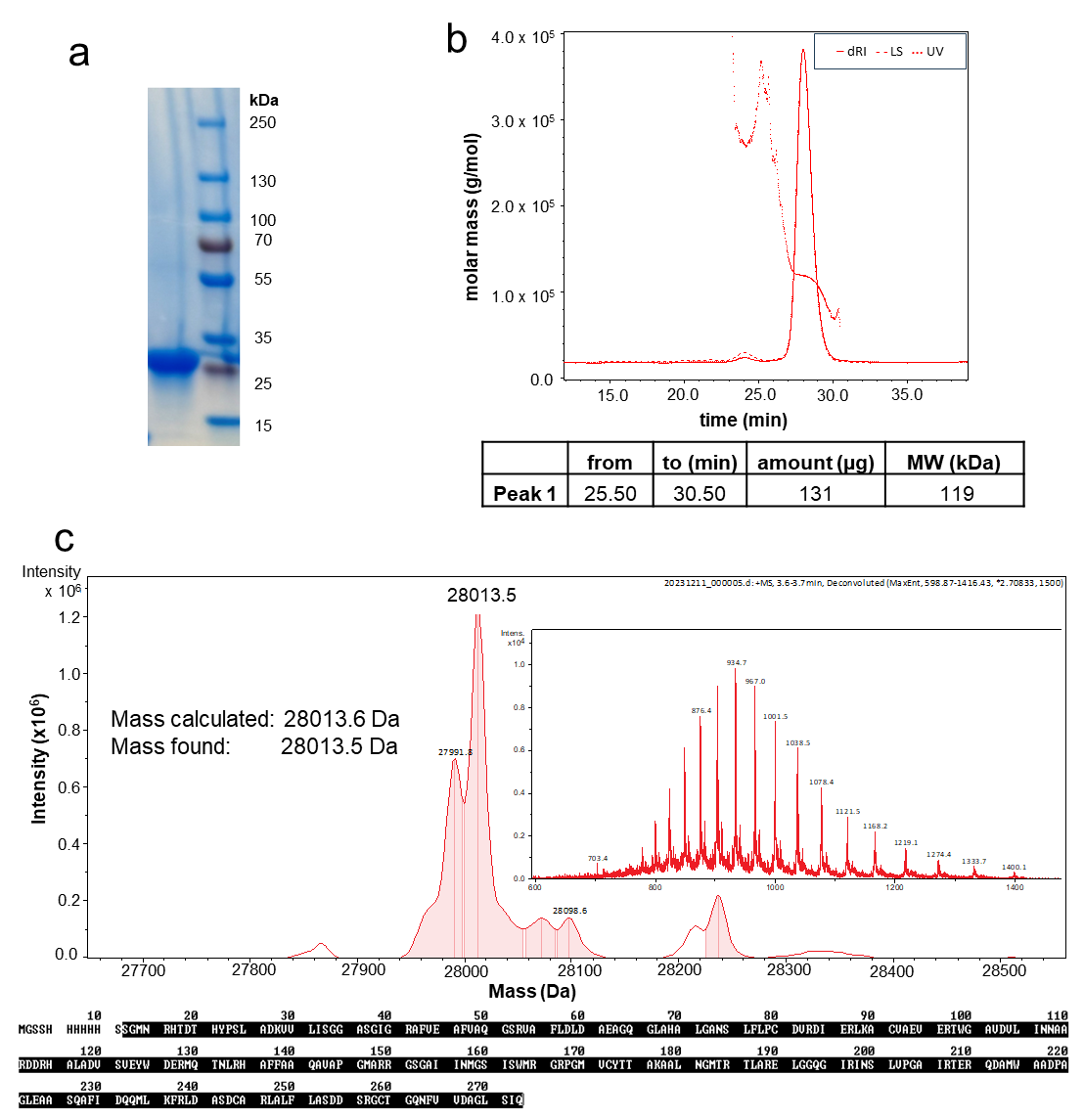
**

**Figure S1**. **Purification and molecular assembly of *Pp*SQDH.** a) SDS-PAGE analysis of purified *Pp*SQDH from *E. coli* after IMAC and size exclusion chromatography (SEC) showing expected MW of ~28,000 Da. b) SEC-MALLS plot reveals the oligomeric state of *Pp*SQDH in solution. UV-trace and an average molecular weight trace (red), calculated from the refractive index and light scattering signal gave mass estimation of 119 kDa, which corresponds to a homotetramer. The area eluted under the major peak corresponds to 131 µg, which comprises >98% of the eluted material confirming homogeneity of the sample. The minor peak at 24 min is about 1.6% of the sample and its estimated MW consistent with a dimer of the tetramers. c) ESI-MS of *Pp*SQDH confirming molecular weight and identity of pure protein.

**
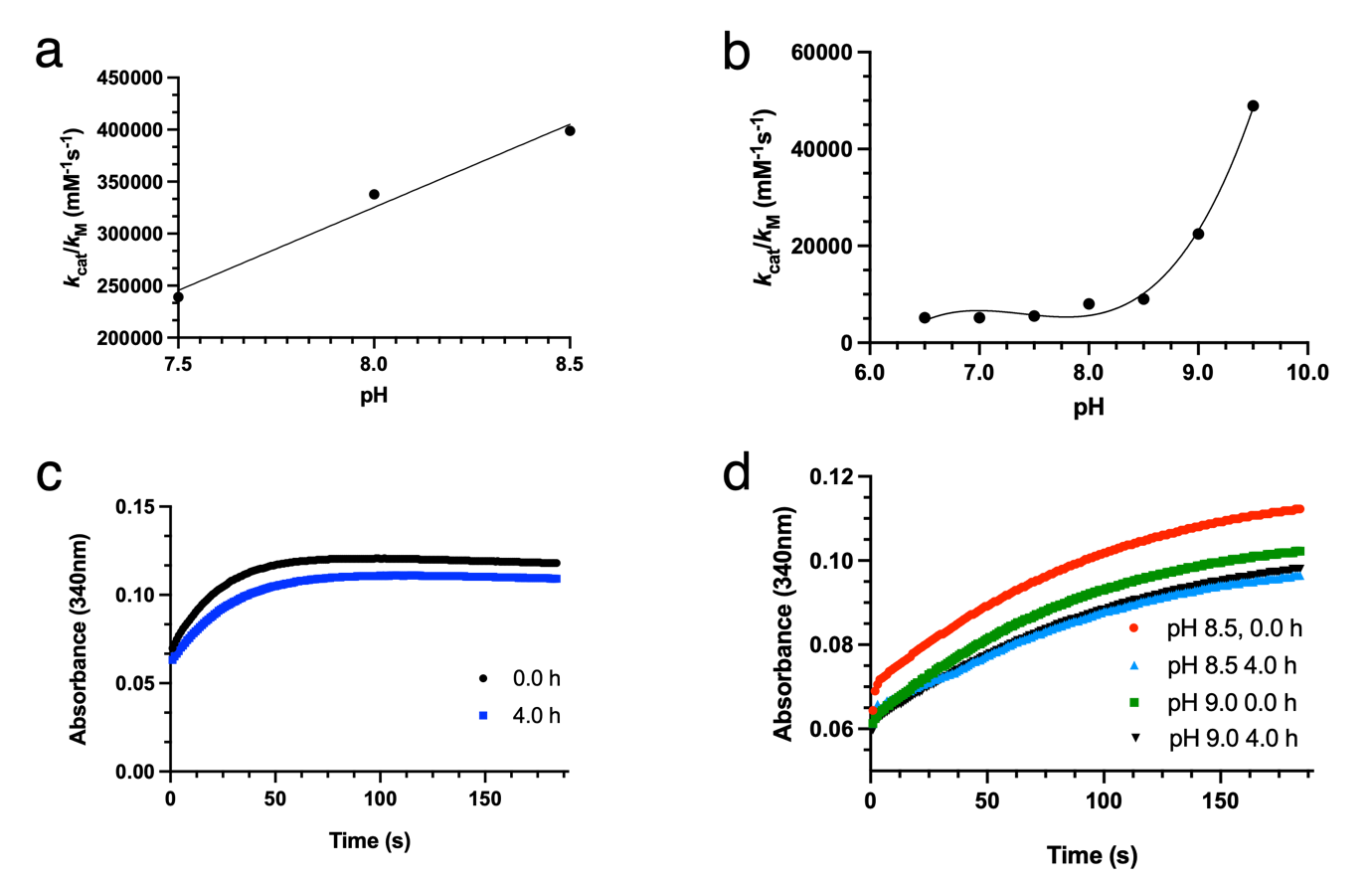
**

**Figure S2.** **pH dependence and stability of *k*_cat_/*K*_M_ of SQ oxidation catalyzed by *Pp*SQDH with tricine and Bis-Tris-Propane buffer.** a) Reactions contained 50 mM tricine (at varying pH), [NaCl] = 150 mM, [SQ] = 0.2 mM, [NAD^+^] = 0.3 mM, and were initiated by addition of [*Pp*SQDH] = 1.4 nM. b) Reactions contained 50 mM Bis-Tris-Propane (at varying pH), [NaCl] = 150 mM, [SQ] = 0.2 mM, [NAD^+^] = 0.3 mM, and were initiated by addition of [*Pp*SQDH] = 13.2 $\mu$M. Rates were scaled in proportion to allow comparison of a and b. c) Stability of enzyme with 50 mM tricine (at pH 8.5), [NaCl] = 150 mM, [SQ] = 0.2 mM, [*Pp*SQDH] = 13.2 $\mu$M and were initiated by addition of [NAD^+^] = 0.3 mM after 0.0 h. d) Stability of enzyme with 50 mM Bis-Tris-Propane (at pH 8.5 and 9.0), [NaCl] = 150 mM, [SQ] = 0.2 mM, [*Pp*SQDH] = 13.2 $\mu$M and were initiated by addition of [NAD^+^] = 0.3 mM after 4.0 h. Stability tests show faster loss of enzyme activity with BTP buffer. Thus, tricine pH 8.5 was chosen as the preferred buffer.

**Figure S3.** **Pseudo-first order kinetics for *Pp*SQDH with G6P.** Michaelis-Menten kinetic analysis with 50 mM tricine buffer (pH = 8.5), [NaCl] = 150 mM, [NAD^+^] = 0.3 mM, [*Pp*SQDH] = 0.48 mM, and varying concentrations of G6P (16–200 mM).

**
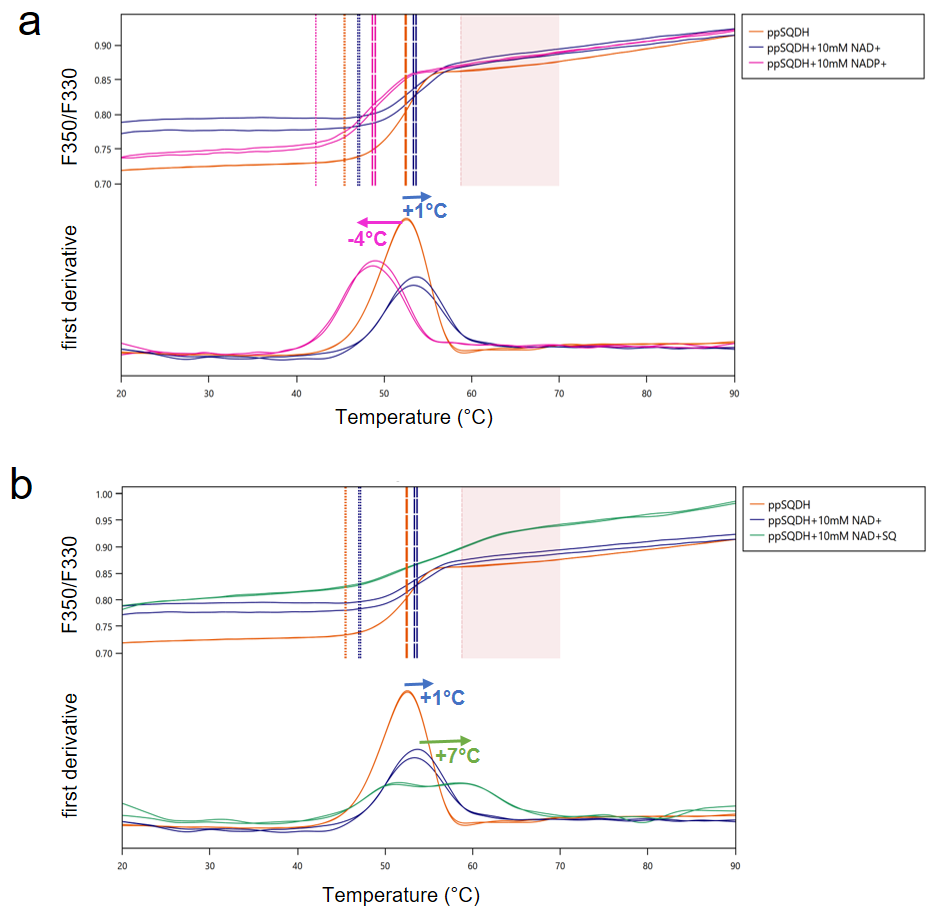
**

**Figure S4. Nano-DSF analysis of *Pp*SQDH.** a) Thermal unfolding profile of *Pp*SQDH showing shift in melting temperature (Tm) of cofactor-free *Pp*SQDH (orange), versus when incubated with 10 mM NAD^+^ (blue) and destabilizing interactions with 10 mM NADP^+^ (magenta). b) Thermal unfolding profile of *Pp*SQDH showing shift in melting temperature (Tm) of cofactor-free *Pp*SQDH (orange), versus when incubated with 10 mM NAD^+^ (blue) and when incubated with both co-substrates, 10 mM NAD^+^ and 10 mM SQ (green).


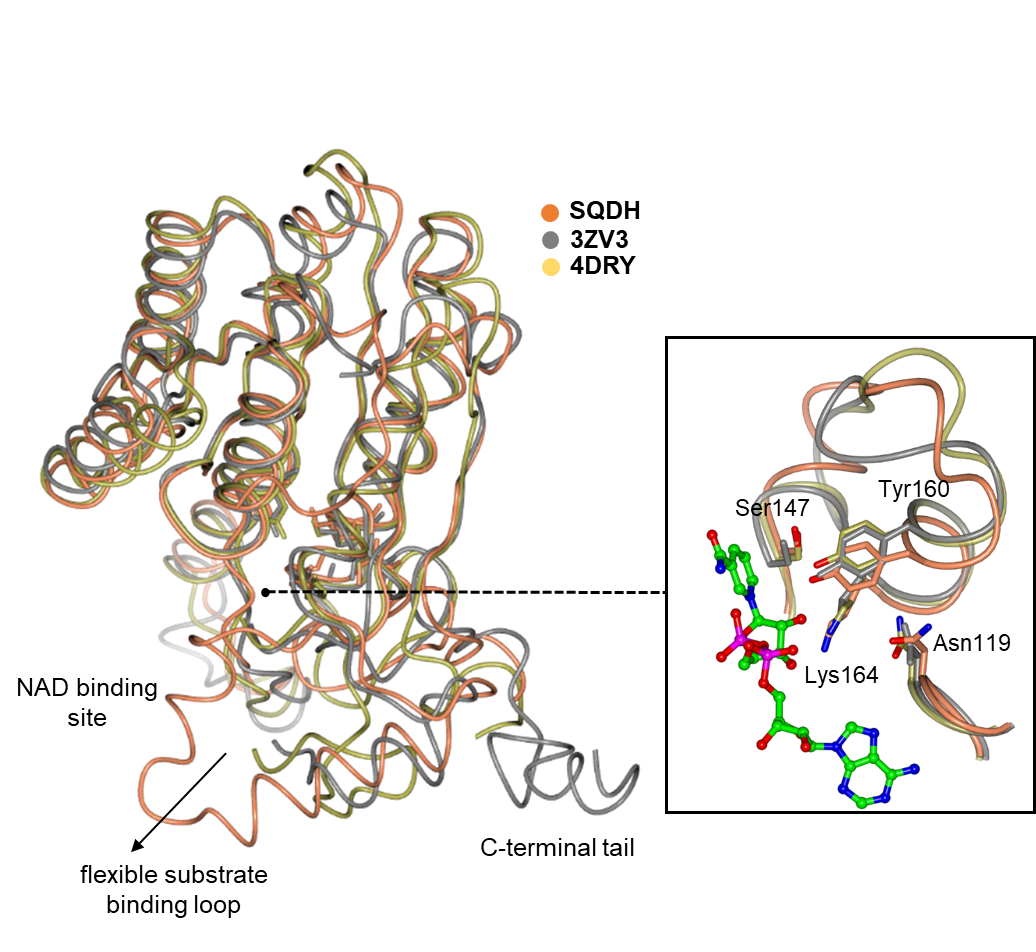


**Figure S5. Superposition of structural homologs of *Pp*SQDH**. Subunit A of *Pp*SQDH (coral) superposed on close structural relatives identified using DALI analysis to show the similarities of the core structure comprising Rossmann fold for binding the nucleotide and the catalytic tetrad Tyr-Ser-Lys-Asn residues (*Pp*SQDH numbering) are shown in the inset. The homologs display differences in the length and conformations of the substrate binding loop. 3-oxo-[acyl-carrier-protein] reductase from *Rhizobium meliloti* (in grey) (PDB ID: 4DRY) andcis-biphenyl-2,3-dihydrodiol-2,3-dehydrogenase from *Pandoraea pnomenusa*. (PDB ID: 3ZV3) are shown.

**Supplementary Table**

**Table S1.** Data collection and refinement statistics. Numbers in brackets refer to data for highest resolution shells.

|  | **apo-*Pp*SQDH** | ***Pp*SQDH•NAD** | ***Pp*SQDH•SQ** |
| --- | --- | --- | --- |
| Data collection | | | |
| Wavelength (Å) | 0.976 | 0.976 | 0.976 |
| Space group | I121 | I121 | I121 |
| Molecules in A.S.U | 2 | 2 | 2 |
| Cell dimensions | | | |
| *a*, *b*, *c* (Å) | 82.73, 57.29, 91.27 | 82.89, 57.02, 91.61 | 82.87, 57.29, 91.98 |
| α, β, Ɣ (°) | 90.00, 93.40, 90.00 | 90.00, 93.45, 90.00 | 90.00, 93.12, 90.00 |
| Resolution (Å) | 63.07-1.70 (1.73-1.70) | 46.95-1.90 (1.94-1.90) | 63.21-1.90 (1.94-1.90) |
| *R*_merge_ | 0.08 (0.88) | 0.10 (0.62) | 0.08 (0.76) |
| *R*_pim_ | 0.03 (0.36) | 0.04 (0.28) | 0.03 (0.34) |
| *I* / σ*I* | 12.80 (2.10) | 10.20 (2.30) | 11.80 (2.10) |
| CC1/2 | 1.0 (0.76) | 0.99 (0.96) | 1.0 (0.81) |
| Completeness (%) | 99.30 (99.80) | 99.60 (99.90) | 97.80 (99.50) |
| Redundancy | 6.70 (6.80) | 6.30 (6.10) | 6.30 (5.90) |
| Unique reflections | 46667 (2440) | 33641 (2154) | 33364 (2182) |
| Refinement | | | |
| Resolution (Å) | 63.07-1.70 | 46.95-1.90 | 63.21-1.90 |
| *R*_work_ / *R*_free_ | 0.2050/0.2380 | 0.2070/0.2420 | 0.2150/0.2400 |
| No. of atoms | | | |
| Protein | 3797 | 3678 | 3681 |
| Ligand/ion | - | 144 | 54 |
| Water | 181 | 95 | 153 |
| *B*-factors (Å^2^) | | | |
| Protein | 32 | 34 | 39 |
| Ligand/ion | - | 26 | 30 |
| Water | 32 | 30 | 41 |
| R.M.S. deviations | | | |
| Bond lengths (Å) | 0.0078 | 0.0073 | 0.0066 |
| Bond angles (°) | 1.6340 | 1.5800 | 1.5740 |
| Ramachandran Plot Residues | | | |
| In most favourable regions (%) | 96.9 | 95.5 | 96.8 |
| In allowed regions (%) | 3.1 | 4.3 | 3.0 |
| Outliers (%) | 0.0 | 0.2 | 0.2 |
| **PDB code** | **9GWU** | **9GWV** | **9GWW** |
